# A New Model for Pore Formation by Cholesterol-Dependent Cytolysins

**DOI:** 10.1371/journal.pcbi.1003791

**Published:** 2014-08-21

**Authors:** Cyril F. Reboul, James C. Whisstock, Michelle A. Dunstone

**Affiliations:** 1 Department of Biochemistry and Molecular Biology, Monash University, Clayton Campus, Melbourne, Victoria, Australia; 2 ARC Centre of Excellence in Advanced Molecular Imaging, Monash University, Clayton Campus, Melbourne, Victoria, Australia; 3 Department of Microbiology, Monash University, Clayton Campus, Melbourne, Victoria, Australia; Tel Aviv University, Israel

## Abstract

Cholesterol Dependent Cytolysins (CDCs) are important bacterial virulence factors that form large (200–300 Å) membrane embedded pores in target cells. Currently, insights from X-ray crystallography, biophysical and single particle cryo-Electron Microscopy (cryo-EM) experiments suggest that soluble monomers first interact with the membrane surface *via* a C-terminal Immunoglobulin-like domain (Ig; Domain 4). Membrane bound oligomers then assemble into a prepore oligomeric form, following which the prepore assembly collapses towards the membrane surface, with concomitant release and insertion of the membrane spanning subunits. During this rearrangement it is proposed that Domain 2, a region comprising three β-strands that links the pore forming region (Domains 1 and 3) and the Ig domain, must undergo a significant yet currently undetermined, conformational change. Here we address this problem through a systematic molecular modeling and structural bioinformatics approach. Our work shows that simple rigid body rotations may account for the observed collapse of the prepore towards the membrane surface. Support for this idea comes from analysis of published cryo-EM maps of the pneumolysin pore, available crystal structures and molecular dynamics simulations. The latter data in particular reveal that Domains 1, 2 and 4 are able to undergo significant rotational movements with respect to each other. Together, our data provide new and testable insights into the mechanism of pore formation by CDCs.

## Author Summary

Pore formation is central to the ability of cholesterol dependent cytolysins (CDCs) to act as important bacterial virulence factors. Secreted by numerous pathogens the toxins assemble into a circular ring and then perforate the target membrane to form the largest self-assembling proteinaceous pores known. In this paper we investigated computationally the conformational properties of the CDC molecule and deduced a new structural model of pore formation and membrane insertion that reconciles all experimental data. The mechanism of membrane perforation by CDCs put forward here reveals concerted and unsuspected domains motion of large amplitude, which conflicts with the currently proposed model. The work presented here procures a plausible structural mechanism of CDC oligomeric transition and furthers our understanding of pore formation by these important toxins.

## Introduction

Cholesterol dependent cytolysins (CDCs) represent a major branch of the CDC/membrane attack complex/perforin-like (MACPF) protein superfamily. Originally identified as virulence factors produced by Gram positive pathogens, CDC toxins have recently been identified in Gram negative bacteria such as *Desulfobulbus propionicus* and *Enterobacter lignolyticus* [1], [2]. Well-studied family members include perfrinolysin O (PFO), pneumolysin (PLY), listerolysin O (LLO), streptolysin O (SLO) and intermedilysin (ILY). A unifying feature of these toxins is the ability to assemble into giant, membrane embedded pores [1]. Pore formation is associated with a variety of toxic functions, including escape from the intracellular phagolysosome (LLO) [3] and the delivery of folded toxins such as nicotinamide adeninedinucleotide-glycohydrolase by SLO [4].

**Figure 1:**
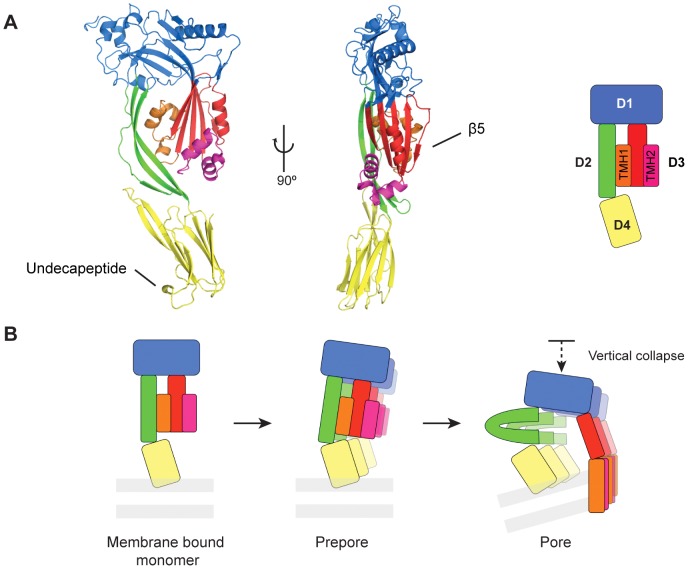
CDC domain organisation and mechanism of pore formation. A. Crystal structure of the archetypal CDC PFO and its schematic representation. Domain 1 is coloured blue, Domain 2 coloured green, Domain 3 coloured red, orange and pink and Domain 4 coloured yellow. Together Domains 1 and 3 form the ‘head’ domain distantly related to the MACPF domain. Specific transmembrane regions include the TransMembrane Helices (TMH) 1 coloured orange and TMH2 coloured pink; the strand β5 and the undecapeptide loop are indicated. B. Current model of CDC pore formation. After the membrane binding event, monomers oligomerize into a ring-like structure (30 to 50 monomers; prepore). Upon formation of the oligomeric pore, both helical clusters insert into the transmembrane bilayer (grey bars) as two β-hairpins (orange and pink) part of a giant β-sheet barrel. Concomitantly Domain 1 is subject to a vertical collapse associated with a proposed “buckling” of Domain 2 (reviewed in [1], [58]).

The structure of CDCs has been well studied. The first crystal structure of a monomeric CDC (PFO) suggested that the molecule comprises four distinct domains. Domains 1 and 3 are non-contiguous regions forming a head region that is linked via Domain 2 to the Ig-like Domain 4 (Figure 1A) [5]. The mechanism of CDC membrane insertion has also been well characterized and mapped to the structure. Briefly, during pore formation two clusters of helices (Transmembrane Helix 1 (TMH1) and 2 (TMH2)) within Domain 3 unwind and insert into the membrane as two amphipathic β-hairpins. Together, Domains 1 and 3 are homologous to the distantly related MACPF proteins [6]. Domain 2, a region unique to CDCs, essentially comprises an elongated three-stranded β-sheet that links the pore forming head domain (Domains 1 and 3) to Domain 4. Finally, Domain 4 contains the determinants for interacting with the membrane, including a key conserved sequence that is important for binding cholesterol [7].

Single particle cryo-Electron Microscopy (SP-cryo-EM), Atomic Force Microscopy and Förster Resonance Energy Transfer (FRET) studies [8]–[10] have provided key insights into the transition from the prepore to the pore structure. Following interaction with the membrane surface *via* Domain 4, CDC monomers assemble into a prepore form. In this conformation, SP cryo-EM data suggest that the conformation of each subunit broadly resembles that seen in crystal structures (i.e. no major conformational change is apparent). Biophysical and microscopy data reveal that following prepore assembly, and in order to form a transmembrane pore, Domains 1 and 3 undergo a significant 40 Å movement towards the membrane surface [9], [10]. Further, the cryo-EM structure of the pneumolysin pore [8] shows that the central four-stranded β-sheet opens, an event that separates Domains 2 and 3. Concomitant with these events, the two small clusters of α-helices TMH1 and TMH2 on either side of the central sheet unwind and insert into the membrane as amphipathic β-strands (Figure 1B).

The conformational changes that surround Domains 1 and 3 are relatively well understood. However, a key question remains about how the prepore form collapses towards the membrane surface. Interpretation of cryo-EM data strongly suggests that Domain 2 “buckles” or “doubles over” itself. However, these data are of low resolution (29 Å) and to date it has not been possible to unambiguously model the position and conformation of Domain 2 [8]. Furthermore, attempts at conformationally trapping Domain 2 to prevent buckling have been unsuccessful [11]. Therefore understanding the structural perturbations that take place in Domain 2 remains central to understanding the mechanism of membrane insertion in CDCs.

Previous crystallographic studies have demonstrated wide variability in the position of the membrane binding Domain 4 with respect to Domains 1, 2 and 3 [12], [13]. It has also been suggested that Domain 2 distortion governs different orientations of Domain 4 [12]. In contrast, a second hypothesis postulates that movement in Domain 4 is entirely attributable to a hinge bending motion located at the Domain 2/4 interface [11], [14]. However, to date, there has been no family-wide description of the regions of rigidity and plasticity of the CDCs.

Here, we characterize the variability between the fifteen available CDC crystal structures and use this information to re-visit the role of Domain 2 in conformational change using the published cryo-EM maps [8] This analysis allowed a novel and methodical molecular model building strategy. Our data suggest that a rotational collapse involving Domain 2 provides the most logical mechanistic model for CDC pore formation with the current available data.

## Results and Discussion

### The CDC monomers: Rigid fragments and regions of deformation

**Figure 2:**
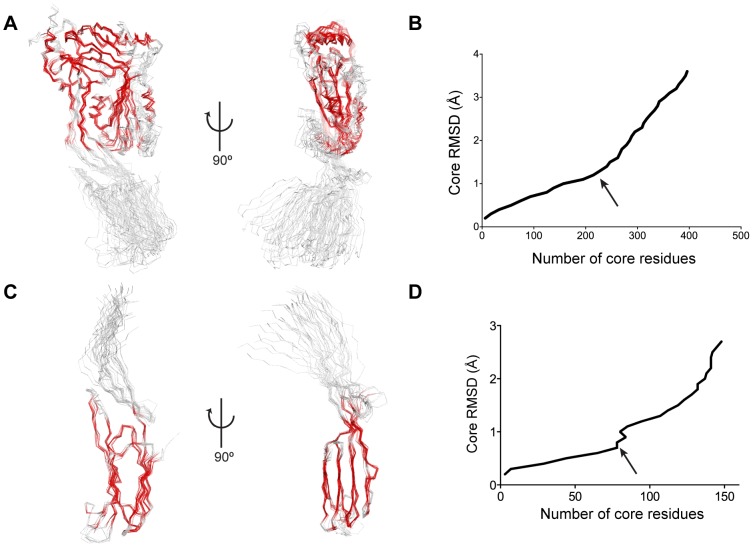
Identification of rigid fragments in CDC structures. A & B. Structural alignment of all CDC structures and corresponding Lesk-Hubbard plot. 3.7 Å Cα-RMSD for 429 conserved positions; the common core at 1.3 Å sieving RMSD (indicated by the arrow) in B in red (228 positions). In Domain 3, the β5 strand and the TMH2 helical bundle are regions of plastic deformation. C & D. Structural alignment of all CDC Domain 2 and 4 structures and corresponding Lesk-Hubbard plot. 1.9 Å Cα-RMSD for 157 residues; common core at 0.8 Å sieving RMSD (indicated by the arrow) in red (78 positions). The undecapeptide and the protruding β-hairpin are regions of plasticity.

To characterise the rigid fragments we performed superposition experiments [15] on all known CDC crystal structures (Table 1). By first aligning the whole molecules we identified a major rigid body consisting of Domains 1, 3 (excluding the TMH2 region) and the upper part of Domain 2 close to Domain 1 and packing against TMH1 (positions 53–56; 81–90; 380–384) (Figure 2A,B). The alignment highlights the structural variability of the base of Domain 2 as well as the variability of Domain 4 orientation across the family (Figure S1). Further, structural alignments of Domains 2 and 4 either separately or together (Figure 2C,D) demonstrate that Domain 4 is to be treated as a rigid body and identify the base of Domain 2 as a region of plastic deformation.

**Table 1:** CDC crystal structures used in this study.

CDC (abbreviation)	PDB ID	Resolution (Å)	Molecules in ASU	Abbreviation used in this study
Perfringolysin O (PFO)	1pfo	2.20	1	PFO I
	1m3j	2.90	2	PFO II A, B
	1m3i	3.00	4	PFO III A, B, C, D
Intermedilysin (ILY)	1s3r	2.60	2	ILY I A, B
	4bik	3.49	2	ILY II A, B
Anthrolysin O (ALO)	3cqf	3.10	2	ALO A, B
Streptolysin O (SLO)	4hsc	2.10	1	SLO
Suilysin (SLY)	3hvn	2.85	1	SLY

ASU stands for asymmetric unit.

**Figure 3:**
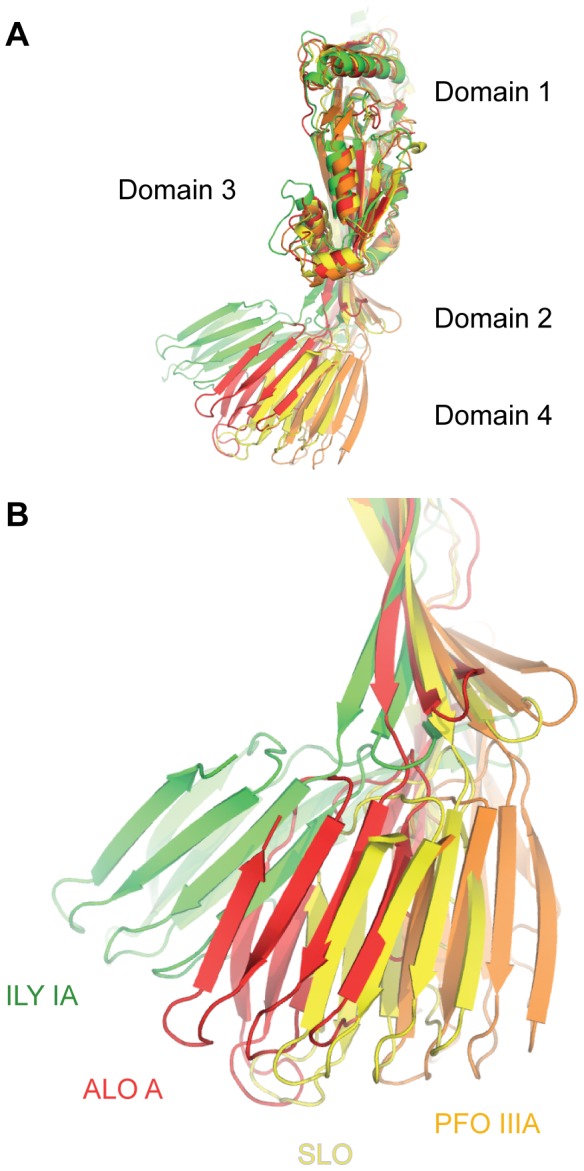
Twist of Domain 2 and its influence on Domain 4 orientation. A. Variations in Domain 4 orientation across the CDC family. Superposition is the same as Figure 2A. Only four representative structures are shown, which cover the entire range of Domain 4 orientation in CDCs. B. Coupling between Domain 2 twist and Domain 4 orientation. Domain 3 is omitted for clarity.

Closer inspection of the structural alignments identified a direct relationship between the deformation of residues in the base of Domain 2 (positions 69–76; 387–390) and the orientation of Domain 4. As the overall twist in Domain 2 β-sheet increases Domain 4 rotates away from the body of the molecule by up to 35**°** (Figure 3). This defines that the orientation of Domain 4 in CDCs is in part attributable to the plasticity at the base of Domain 2.

### The conformational properties of Domain 2 in PFO

**Figure 4:**
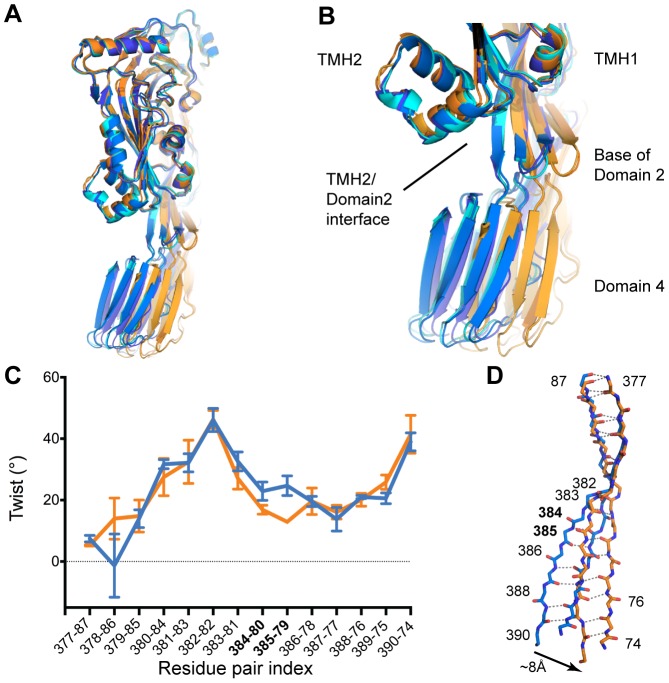
PFO Domain 4 and twist of Domain 2. A. Variations in Domain 4 orientation for the PFO structures. Superposition is the same as in Figure 2A. Only five structures are shown for clarity: PFO I (blue); PFO IIB (dark blue); PFO IIID (cyan); PFO IIIA (orange); PFO IIIC (light orange). B. Coupling between PFO Domain 2 twist and Domain 4 orientation. Domain 3 is omitted for clarity. C. Twist values as a function of amino acid pairs for the continuous strands forming Domain 2. The seven crystal conformations are classified in two groups: conformations with partial loss of Domain 2/4 interface (interface ∼425 Å^2^; PFO IIIA & IIIC) in orange and all the others in blue (interface ∼605 Å^2^; Table 2). Minimum and maximum values are displayed for each group. D. Representative mainchains of Domain 2 β-sheet in stick representation. Blue: PFO I; orange: PFO IIIA. Superposition is the same as in Figure 2A. Numbers indicate amino-acid positions, dashed lines indicate mainchain hydrogen bonds.

We next investigated the deformation in PFO Domain 2. This is made possible by the availability of multiple structures that allow us to define the conformational space accessible to one molecule. We identified seven conformations derived from three crystal forms (Table 1 and [16]). In two conformations (PFO IIIA and C) the interface between Domains 2 and 3 is partially disrupted. PFO IIIA and IIIC conformers display a loss of contacts between the TMH2 region and Domain 2, corresponding to a loss of ∼180 Å^2^ (30%) surface area. This loss of surface area is associated with an increased distance between the pair of residues Ser287 (TMH2) and Glu388 (Domain 2) compared to the other five conformers (Table 2 and [16]). Notably these contacts all involve amino acids located at the base of Domain 2 (Ile76, Ser386 & Glu388) that we have identified as a region of plasticity (Figure 4D).

**Table 2:** Domain 2-Domain 3 interface features and Molecular simulations performed.

Molecule/Isoform	Domains 2/3 Interface Area (Å^2^)	TMH2/Domain 2 Cα distance (Å)^a^	MD simulation performed^b^
PFO I	578	4.8 (S287-E388)	2 (1)
PFO IIA	609	5.2	-
PFO IIB	641	5.3	-
**PFO IIIA**	**416**	**10.2**	1 (0)
PFO IIIB	592	5.5	1 (1)
**PFO IIIC**	**430**	**8.7**	1 (1)
PFO IIID	611	5.4	-
ILY IA	577	6.1 (K313-V413)	2 (1)
ILY IB	610	6.3	-
ILY IIA	612	6.3	-
ILY IIB	590	7.1	-
ALO A	651	5.6 (K299-T399)	2 (0)
ALO B	679	5.6	-
SLO	595	5.2 (K357-T458)	2 (0)
SLY	593	6.4 (S284-S383)	2 (1)

a Pairs of residues with corresponding numbering are given in brackets.

b The number of simulations where transitions of the Domain 2/TMH2 interface occurs is given in brackets. Except for PFO I MD simulations, only one simulation is presented in this work.

Although it has been identified that the loss of contacts at the Domains 2/4 interface is associated with distortion of the elongated Domain 2 β-sheet [16], little characterization of this region has been performed. Consequently we focused on the deformation in PFO Domain 2 by measuring the twist of the β-sheet in terms of inter-strand pairing of amino acids upon partial loss of this interface (Figure 4C,D). The twist values represented in Figure 4C show moderate differences as they mostly overlap and are characterized by two peaks at positions 382 (high twist central to the sheet) and 390 (C-terminal region of the sheet) (Figure 4D). However, we note a reduced twist in conformations with a weaker interface (orange) with differences of 6° and 11° at two consecutive positions: Thr384 and Thr385. A lower twist of the sheet at these positions immediately comprised between the major central twist and residues involved in the TMH2-Domain 2 interface leads to a significant shift of ∼8 Å of the C-terminal segment of Domain 2 (Figure 4D). This is indicative that the partial loss of the interface is structurally coupled with un-twisting of Domain 2.

**Figure 5:**
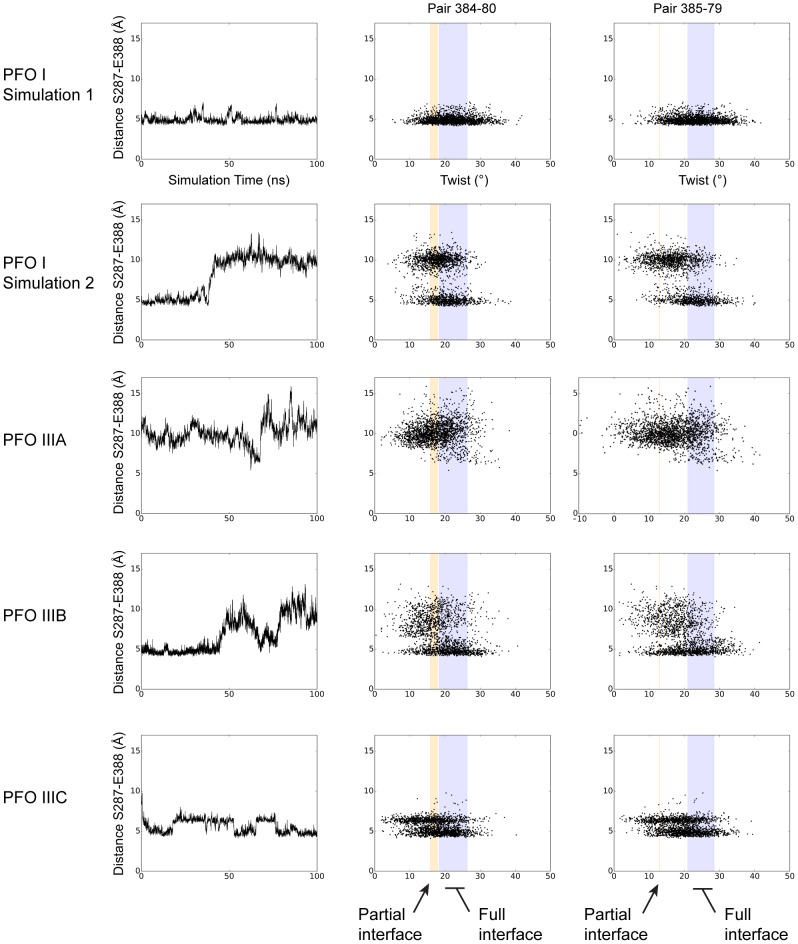
Plasticity and deformation of Domain 2 from PFO MD simulations. Analysis of the different MD simulations for PFO I, PFO IIIA, PFO IIIB, PFO IIIC (indicated at the left of each panel). Left panel: distance representative of the Domain 2/TMH2 interface between residues 287 and 388 (see also Table 2). Centre and right panels: values of twist at positions discussed in the text and illustrated in Figure 4C plotted versus the distance between residues in left panel. The coloured vertical bars correspond to the range of twist values derived from the analysis of the PFO crystal structures (Figure 4B). The pairs of residues considered are indicated at the top of the figure.

As crystallographic packing artifacts may limit our structural analysis we performed a series of molecular dynamics (MD) simulations starting from conformations with either a full (PFO I×2, PFO IIIB×1, summarized in Table 2) or partial (PFO IIIA×1; PFO IIIC×1) Domains 2/TMH2 interface. We observed that the molecule has the ability to transition between full and partial interface (PFO I Simulation 2, PFO IIIB, Figure 5 and Videos S1, S2, S3, S4, S5). Conversely PFO IIIC was able to transition from a partial interface to a full interface. Associated with the fluctuations of the interface, we observed a clustering of twist values at the positions outlined by our structural analysis (Thr384-Arg80 and Thr385-Glu79, Figure 4C, Table 2). An increase in the distance between TMH2 and Domain 2 (i.e. from full to partial interface) is associated with a decrease in the twist values (Figure 5). We therefore conclude from MD simulations that PFO has the potential to fluctuate between discrete states independent of crystal packing. In addition, structural analysis of the MD simulations identified a common pattern whereby the partial loss of the interface is structurally coupled with un-twisting, or straightening, of Domain 2.

**Figure 6:**
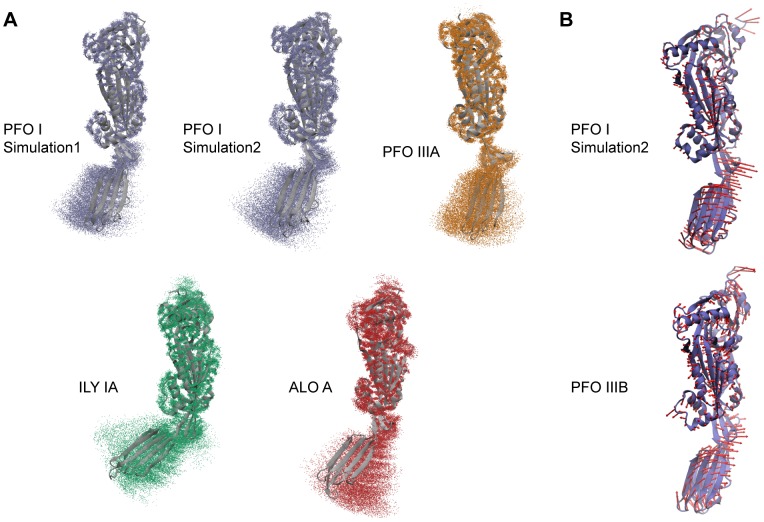
Flexibility of Domain 4 with respect to Domains 1–3 in MD simulations. A. Each panel corresponds to an MD simulation. The starting conformer (cartoon representation, grey) is indicated at the left of the molecule. Cα positions taken from snapshots of the simulations are represented as dots after alignment on Domains 1–3. B. Arrows (red) indicate the directions of motion of the third slowest mode (scaled to 3.5 Å for clarity), which best describes the increased distance between Domain 2 and TMH2. The starting conformer is indicated at the left of its cartoon representation. The reasonably small fractional variance explained by the modes (11% (PFO I) and 8% (PFO IIIB), collectivity of 0.34 and 0.45 respectively) identifies a more localized nature of the motion. This suggests that such movement participates to, or enhances, the observed flexibility of Domain 4.

Next we analyzed our MD simulations in terms of the positions adopted by Domain 4. In all simulations performed, Domain 4 exhibited a large range of orientations (Figure 6A, Figure S2). Principal component analysis of the MD simulations identified such domain movement as the slowest ‘breathing’ mode of motion with a minimum of the total variance explained of 40% and a collectivity of typically 0.6 across all simulations. Furthermore, in simulations where the Domain2/TMH2 interface transitions between full and partial states (PFO I simulation 2, PFO IIIB) we found that a single mode (the third slowest mode in both cases) best described the departure of the base of Domain 2 from TMH2. Notably, this mode also encapsulated accompanying motion of Domain 4, rotating away from the body of the molecule (Figure 6B). Thus, MD simulations further support that the orientation of Domain 4 in PFO can be ascribed to the plasticity at the base of Domain 2.

### The conformation properties of CDCs

To extend our investigation of the conformational properties of CDCs we performed additional MD simulations of other members of the family including intermedilysin, suilysin, anthrolysin and streptolysin. The major observation is the consistent flexibility of Domain 4 with respect to domains 1–3, similar to the flexibility observed for PFO (Figure 6A, Figure S2B, Videos S6,S7).

Secondly, for ILY IA and to a smaller extent for SLY simulations we observed a reduction of twist where the Domain 2/TMH2 interface is partially lost (Figure S2A). This correlates with the observations from the PFO simulations. We also noted that partial loss of the interface was associated with a similar decrease in twist at nearby positions (ILY IA (105–412) and SLY (73–383), not shown). This data suggests the torsion of the β-sheet may be subtly modulated in a toxin-dependent fashion.

Overall these data, although non-exhaustive, support that two characteristics emerge as unifying features across the CDC family: the substantial relative flexibility of Domain 4 and the ability of Domain 2 to straighten upon weakening of the Domain 2/TMH2 interface.

Taken together, our cross-comparison of all available CDC structures and MD simulations analysis allow us to define the conformational properties of the molecule in the monomeric form. Domain 2 wraps around TMH1 and the base of TMH2 and most likely prevents their premature release and aggregation of the molecule (Figure 1A and as demonstrated in the case of LLO [17]). Our data suggest that preservation of the interface of TMH1 and TMH2 with Domain 2 is accompanied by conformational torsion in Domain 2 and twisting of the elongated sheet. We suggest that release of this interface, an early and critical step of membrane insertion [18], results in the straightening of Domain 2.

Domain 2 has been first proposed to undergo some conformational change after loss of TMH1/2 contacts in order to account for the 40 Å vertical collapse observed upon prepore-to-pore transition [9]. Tilley and coworkers [8] hypothesized that one way to account for this collapse was for the triple stranded β-sheet of Domain 2 to fold sharply in half. Here, and in contrast to this idea, our combined structural analysis and MD simulations suggest that Domain 2 has the propensity to straighten upon the loss of TMH1/2 contacts. Moreover, we argue that an energetic requirement to preserve inter-strand hydrogen bonds and local packing in anti-parallel β-sheets favours continuous deformation of the Domain 2 region rather than a major collapse [19]. Given this analysis, we re-visited the conformational states of prepore and pore in CDCs with improved knowledge of their conformational properties.

### The domain architecture of the prepore oligomer pre-exists in the isolated monomer

**Figure 7:**
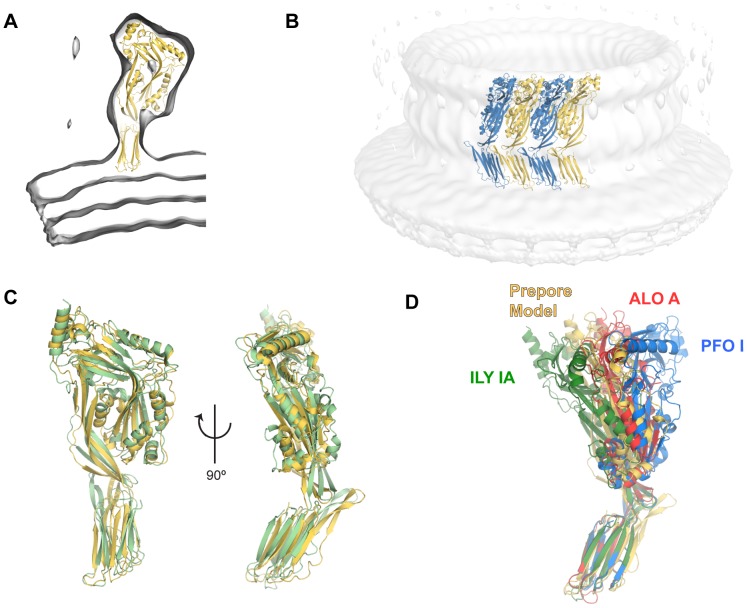
Prepore conformation of the CDC molecule. A. View of the CDC monomer in the prepore conformation within the cryo-EM map (transparent surface). B. Overall oligomeric arrangement within the cryo-EM map. Only the tetramer used in the modeling is shown (see Methods). C. Structural alignment of the ILY IB conformation (pale green) to the prepore conformation (pale orange) (4.4 Å; 448 positions). D. Prepore conformation in the context of representative CDC crystallographic structures.

Next we examined the conformation of the PLY monomer within the prepore oligomer. We modeled the prepore conformation within the available cryo-EM map with 31-fold circular symmetry (C31) [8] (Figure 7A,B; detailed in Methods). After the flexible fitting step the cross-correlation coefficient (CCC) for the oligomer improved from 0.57 to 0.61. The structural transitions accompanying the assembly of the prepore are well described. First the conformationally labile β5 strand rotates away from the β4 strand leaving its edge exposed to the formation of mainchain hydrogen bonds with the β1 strand of an adjacent monomer [20]. Our model takes this structural change into account. The β5 strand is modeled here as a short helix by analogy with the structurally equivalent position in the complement component C6 [21] (Figure S3), a member of the distantly but structurally and functionally related MACPF family [6], [22]. Secondly, the oligomer transitions to a SDS-resistant prepore upon the formation of specific β1–β4 contacts [20], [23]. Our prepore model displays some, but not all, oligomeric β1–β4 mainchain hydrogen bonds compatible with the pattern later displayed by the pore form [24], [25] (Figure S4).

In agreement with previous modeling we find that the prepore conformation can be explained solely by a tilt (∼40**°**) of Domain 4 with respect to the long axis of the molecule [8] (Figure 7B,C). Such orientation is supported by the determined solvent exposure of amino acids of Domain 4 [9] (Figure S5). Based on our previous structural and MD simulations analyses we hypothesize that this minor domain rearrangement may be attributable to the intrinsic flexibility of Domain 4 and deformation of Domain 2, although the low resolution of the cryo-EM data (28 Å) prevents further interpretation. In addition, the relative orientation of Domain 4 in the prepore conformation is broadly similar to the crystallographic conformation of ILY IB (Figure 7C), and within the range of observed CDC crystallographic conformations (Figure 7D). Stereochemical features of the model are reported in Table 3. Therefore this indicates that the CDC monomer in the prepore form adopts an arrangement of domains readily accessible in the soluble form, which is conformationally trapped upon oligomerization.

**Table 3:** Stereochemistry indicators for the atomic models.

Indicator	Prepore		Pore		
	Initial^a^	Final	Initial	Final	Minimized
Clashscore	0.37	0.00	1.69	0.00	0.00
Poor rotamers (%)	2.08	6.12	1.77	7.59	4.28
Ramachandran outliers (%)	1.02	4.28	1.23	2.68	2.14
Ramachandran favored (%)	93.68	91.43	94.87	89.88	91.97
Cβ violations	0.84	0.68	0.51	1.24	0.39
Bad bonds (%)	0.00	0.00	0.01	0.01	0.00
Bad angles (%)	0.34	0.90	0.35	1.49	0.43

Stereochemistry indicators are as reported by Molprobity v4.1 [59].

a Values are only reported for the model with optimal CCC.

To validate this prepore model a suggested site of interaction would be between strand 2 of Domain 2 of one molecule with the TMH1 of the adjacent molecule (respectively Thr86-Ser88 and Lys201-Asn205, PFO numbering). This could be performed using either disulphide bond formation or short crosslinkers. Disulphide bond formation experiments have successfully characterized Domain 3 oligomeric interactions in PFO [24].

### A novel domain and subdomain re-organization in the pore form

**Figure 8:**
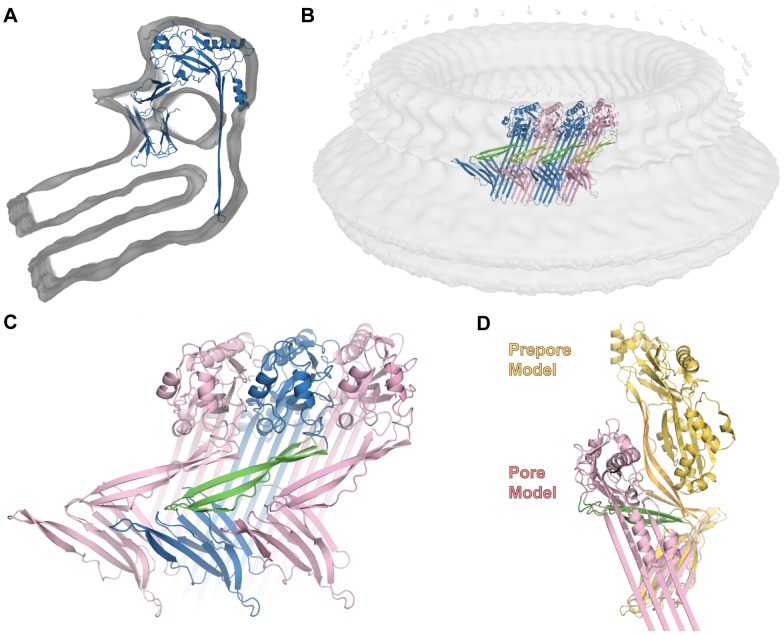
Pore conformation of the CDC molecule. A. Cut view of the CDC monomer in the pore conformation within the cryo-EM map (transparent surface). B. Subunits arrangement in the pore (cut view). The tetramer shown is the symmetrically modeled tetramer (see Methods). Subunits have alternate colouring with Domain 2 coloured green. C. Domains 2 arrangement in the pore viewed from outside of the ring. A Domain 2 is highlighted in green sandwiched by two adjacent monomers. D. Prepore and pore conformations aligned on Domain 4. Prepore Domain 2 is in orange; pore Domain 2 is in green.

Finally we investigated the conformation of CDCs in the pore form to address how CDCs change conformation particularly with respect to Domain 2. Our CDC pore model is presented in Figure 8. Domain 1 can be fitted intact (see Methods, CCC for the individual domain of 0.70) into the cryo-EM density in agreement with Tilley et al. [8]. Together with Domains 1 of adjacent subunits the domains exhibit packing similar to the prepore complex. TMH1/2 are entirely restructured from bundles of α-helices to a giant transmembrane β-barrel, concomitantly with the opening of Domain 3. It has been established that the amino acids forming the β-barrel adopt a novel β-barrel architecture specific to CDCs [24], [25]: the membrane-embedded β-hairpins adopt a 20° tilt to the axis normal to the membrane (Figure 8B). This departs from a pore model where the β-hairpins are proposed to stand perpendicular (0° tilt) to the membrane surface [8]. The modeled β-barrel is in full agreement with the experimentally established amphipathic pattern of the membrane-spanning PFO β-hairpins [26], [27] (CCC of 0.57, Figure S6).

The position of the membrane binding Domain 4 is readily identifiable in the density [8] with only the region surrounding the undecapeptide loop inserting into the upper leaflet of the membrane bilayer [28], [29]. Domain 4 loosely packs against its adjacent counterparts as demonstrated [29]. Its orientation, tilted but not lying on the membrane surface, is further supported by the pattern of solvent exposure of amino acids distributed on the domain's surface [29] (CCC of 0.48, Figure S7).

Taken together, our structural and MD analyses suggest that Domain 2 does not favour the proposed bending [8]. Instead our data suggest that the β–sheet Domain 2 simply untwists and rotates with respect to Domain 4. Motivated by this finding we modeled the elongated β-sheet without altering its structural integrity (see Methods). We found that Domain 2 can be fitted in the density linked to Domain 1 and the Domain 4 of the adjacent monomer (clockwise when viewed from the top; Figure 8C). This dramatic sideways rotation has not been postulated to date, yet it provides a good fit of the domains within the cryo-EM maps. The Domain 2 region in our model has a CCC of 0.52 with no clashes observed between residues, which is excellent for this resolution of cryo-EM data. This is an improvement on the existing unrefined model, which has a CCC of 0.45 for the Domain 2 region (PDB ID 2bk1). Following the flexible fitting step the 38-mer exhibited a best CCC value of 0.67, an improvement on the initial 0.62.

In support of this model we investigated the Domain 2 boundaries with Domain 1 and Domain 4. Our new model preserves the hydrophobic Domain 2/4 interface, with Domain 2 linked to Domain 4 by a glycine linker. There is an introduction of a kink of ∼40° at the Domain 1/2 interface. The Domain 1/2 interface is constituted by three mainchain covalent links and contains no secondary structure elements or specific contacts. In the pore conformation, Domain 2 orientation is at a ∼25**°** angle to the bilayer surface and extends the range of orientations observed in crystallographic structures (Figure 8D, Figure S8). Our model also suggests that the orientation of Domain 2 is constrained by the packing of its adjacent counterparts with the possibility of mainchain parallel hydrogen bonds between positions 54–56 and 384–386 (PFO numbering) of an adjacent monomer. In analyzing conformations fitted at such resolution (29 Å) it should be noted that the predicted interaction is indicative of the close proximity of individual Domains 2 in the pore form (Figure S9). Therefore experiments designed to test this hypothesis should take this aspect into account and may include the use of techniques such as FRET, disulfide bond formation and chemical crosslinking. If strands of Domain 2 are close enough to establish parallel hydrogen bonds then the close proximity can be tested by formation of disulfide bonds. Alternatively in the case of less intimate contacts, chemical crosslinking would be more appropriate. We suggest that probing the Domain 2 oligomeric contact is well suited to distinguishing between the ‘buckling’ model and our proposed model of CDC pore assembly.

To assess the stability of our pore model the fitted conformation was energy-minimized and subjected to a brief MD simulation in a membrane bilayer environment and free of all constraints. After 15 ns of simulation the assembly reached a plateau at 4.4 Å over the last 5 ns (Figure S10). The general subunits arrangement remained stable with little deviation from the initial conformation. Minor structural deviations included a difference in the orientation of Domain 4 as well as its penetration into the membrane bilayer. Since the details of its contacts with the bilayer are currently lacking slight deviations are not unexpected. We also noted in the monomer situated at the clockwise end of the tetramer an increased divergence of Domain 2 and the transmembrane Domain 3 (Figure S10). Given their positions at one extremity of the assembly we concluded that this is likely to be attributable to the lack of explicit circular symmetry in our setup (see Methods) and the absence of buttressing/specific contacts with the adjacent monomer. We found the MD simulation demonstrates the overall stability of our pore model and reflects the quality of its stereochemistry (Table 3).

### Conclusions. Prepore-to-pore transition: A new model of pore formation

This study has also allowed us to map the rigid bodies present in CDCs and their spectrum of flexibility relative to each other. Following this analysis we have revisited both prepore and pore conformations employing available cryo-EM data.

Importantly we find the pore conformation can be modeled without potentially energetically costly restructuring of Domain 2. Instead our modeling suggests that simple domains rotations can account for the well-documented CDC vertical collapse [8]–[10].

**Figure 9:**
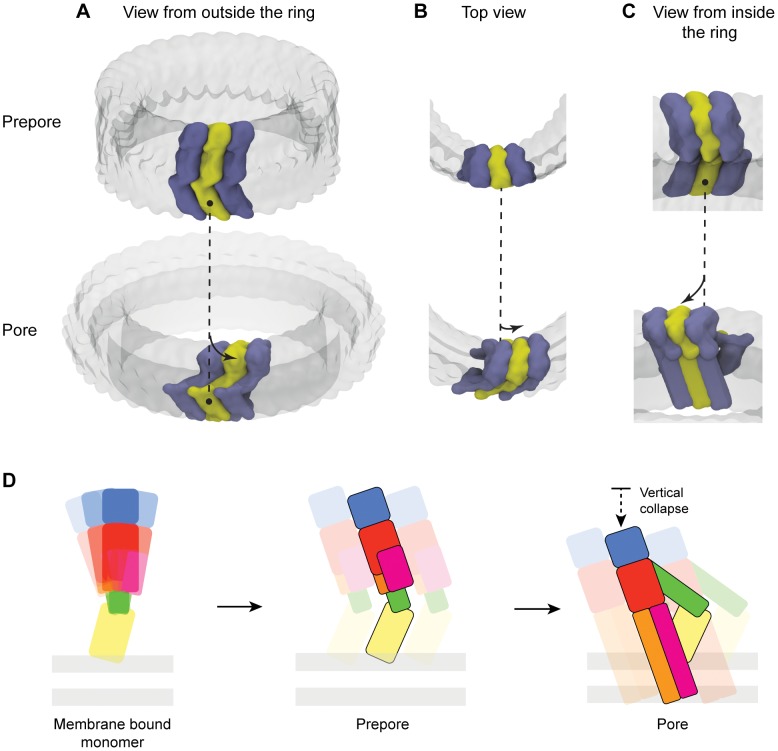
Orchestrated domain movement and proposed mechanism of pore formation. Schematic representation of the proposed new model of prepore (top row) to pore (bottom row) transition shown from three point of views: A) from outside the ring formed by the oligomeric complex, B) Top view and C) from inside the ring. The dashed line symbolizes the position of Domain 4 in the prepore complex. The arrows symbolize the concerted movement experienced by the globular head domain (Domains 1 & 3) upon membrane insertion. Alternate monomers are represented with alternate colors; only three monomers are displayed for clarity; remaining monomers are represented as a transparent surface. D. Schematic representation of proposed CDC pore formation. The monomer displays flexibility in the orientation of Domains 1–3 versus the membrane binding Domain 4. Upon self-oligomerization into the prepore complex the monomer is trapped in a monomer accessible conformation. Upon membrane insertion, the orientation of Domain 2 flattens with respect to the membrane surface. This is accompanied by a vertical collapse of Domain 1 and 3, which brings them closer to the bilayer surface and allows insertion of TMH1/2 as β-hairpins. Colours are identical to Figure 1.

In addition, both our prepore and pore conformations define a pathway for the most logical mechanism of pore formation. Only a coordinated vertical collapse together with rotations of Domains 1/3 (∼10°) is compatible with the extent of Domain 2 rotation (∼60°) from a nearly perpendicular to the membrane surface conformation (prepore) to nearly parallel (pore). Furthermore, the oligomeric packing and specific contacts established in the prepore form [23] are likely to impose the constraints that result in a downward spiral rotation of Domains 1/3 (counter-clockwise rotation corresponding to a monomer and vertical collapse; Figure 9A–C) within the entire oligomeric assembly. This movement defines an unprecedented and orchestrated global motion whereby the prepore transitions to the pore form by rotation of Domain 2 of all subunits, which brings the CDC head domains (Domains 1/3) closer to the membrane surface.

Interestingly, a recent study on aerolysin proposed that a swirling-like motion is central to the mechanism of pore formation by this toxin. While aerolysin and the CDCs are not related, the mechanism we propose is somewhat mechanically analogous [30].

Thus, to conclude, we propose that CDCs achieve pore formation by employing large, concerted domains rotations (schematically summarized in Figure 9D). Our work supports a new model of membrane insertion for CDCs in considerable departure from the currently accepted model. This mechanism presents mechanical similarities to other β-pore forming toxins and a new, testable model of pore formation for CDCs.

## Methods

### Structural alignments and analysis

The identification of common substructures from structural alignments is a powerful approach that allows us to extract the rigid fragments and conformational properties [15], [31] of the CDC molecule. The method provides a standardized way to identify common structural cores of homologous proteins (the “sieving” procedure) [32], [33] although it cannot unambiguously distinguish between conformational change and structural divergence. It is, however, important to note that analysis of structures of one protein, and/or homologous proteins, determined in different conditions captures the conformational features across a family [15], [34]–[36].

All structural alignments were performed with Mustang-MR [37] with domains definition as reported by Rossjohn et al. [5]. Examination and analysis were undertaken using Prody 1.4 [38], Pymol 1.3 [39] and VMD 1.9 [40]. β-sheet twist values are the angles between mainchain vectors of residues in an inter-strand pair and were calculated following Ho and Curmi [41]. Accessible surface areas are as reported by PISA [42]. MD simulations were analyzed with VMD and Prody. PCA were performed with Prody employing 10 K snapshots collected every 10 ps (Cα coordinates).

### Monomeric CDC molecular dynamics simulations

Initial conformations and MD simulations performed are reported in Table 2. In all cases topologies were built and solvated using *teLeap* [43] and the Amber ff99SB force field [44]. MD simulations employed truncated octahedron water boxes (TIP3P, 12 Å padding), sodium and chloride ions were added to charge neutrality. The systems were typically comprised of 164,000 to 171,000 atoms. Temperature was maintained at 300 K using Langevin dynamics with a damping constant of 5 ps^−1^. Pressure was maintained at 1 atm with a Nosé-Hoover-Langevin piston and Periodic Boundary Conditions (PBC) were used. An integration time step of 2 fs was used, short-range forces and long-range electrostatics were calculated every time step. Non-bonded interactions employed a 10 Å cut-off and long-range electrostatic forces were computed by the particle-mesh Ewald (PME) summation method (grid spacing smaller than 1 Å). All systems were subjected to equilibration steps with harmonic restraints first applied to all heavy protein atoms (100 ps, 1 fs time step for this step only), followed by restraints applied only to mainchain atoms (250 ps) and finally Cα atoms (500 ps). 100 ns MD simulations were then produced and analyzed after typically removing the initial 2 to 3 ns. All the simulations were conducted with NAMD v2.9 [45].

### Prepore model

To obtain initial models of the prepore conformation we first performed rigid body docking into the cryo-EM density of representative CDC crystallographic structures in order to leverage the conformational variability highlighted in our analysis.

Four crystallographic structures were used: PFO I, PFO IIIA, ILY IA, ILY IB (cf. Table 1). The PLY sequence (UniProt ID: Q04IN8) was first threaded onto each structural template employing Modeller 2.11 [46] and amino acids corresponding to β5 were simply discarded. The coordinates of the loop at positions 95 to 101 (PFO numbering) were also discarded as the corresponding amino acids were found to produce steric hindrance upon oligomeric assembly. The coordinates were then docked using Situs [47] into the cryo-EM map reconstruction of the PLY prepore (EMDB ID: 1106) with the density of the membrane discarded for this step only as including the bilayer density produced unrealistic placements. The docked individual subunits displayed CCCs ranging from 0.66 to 0.76 (truncated map) and 0.63 to 0.71 (no truncation).

Secondly, for each template the docked structure with the highest CCC was then replicated with C31 symmetry and the positions and orientations of all subunits refined against the density with Situs. Finally, four consecutive subunits (referred to as tetramer in the following) were selected and the missing loop modeled with Modeller, with amino acids corresponding to the β5 region modeled as an α-helix (see text). This led to the production of five initial PLY conformations with a best CCC of 0.57.

Each tetramer was then subjected to a step of flexible fitting into the cryo-EM density with C31 symmetry restraints (see relevant section). A total of 12 models were thus produced with an average root mean square deviation (rmsd) of 2.4±0.9 Å and an average CCC of 0.60±0.01. Although small differences in orientation of the domains were observed (as reflected by the rmsd) all models presented the same domain architecture (discussed in the text). Cα coordinates of the model with optimal CCC (0.61) are provided as Dataset S1.

### Pore model

Domain 1 was initially placed manually in the cryo-EM density (EMD ID: 1107; C38 symmetry; 29 Å resolution) and its position refined locally in the presence of symmetric subunits employing Chimera 1.8 [48]. Here we modeled Domain 3 as a β-barrel with architecture *S* = *n*/2 as detailed in our previous work [25]. Domain 1 orientation was then adjusted to satisfy both a reasonable fit to the density as well as the distance constraints from the four covalent bonds linking Domain 1 and the four β-strands forming the β-barrel (Domain 3).

Before refinement into the cryo-EM density the altitude of Domain 4 was adjusted so as to place the amino acids identified as exposed to solvent and buried in the membrane [29] (Figure S6).

Domain 2 was placed manually in the density without modifications to its internal structure, consistent with the conclusions of our structural analysis. Its initial placement also satisfies the distance constraints imposed by the covalent bonds to Domain 1 and 4 and a reasonable fit to the cryo-EM map.

Our pore model was built as a tetramer with C38 symmetry. Initial coordinates of PFOIII-A were employed with the PLY sequence threaded. Initial positions of all domains were adjusted to remove inter-subunit steric clashes. Furthermore, the initial coordinates were perturbed by 1° clockwise and anti-clockwise rotations around the pore axis thus producing three starting points for the flexible fitting step. The best CCC was 0.62 for the initial conformation.

The three tetramers were subjected to a step of flexible fitting into the cryo-EM density with C38 symmetry (see relevant section). Final average rmsd was 1.4±0.4 per monomer and the average CCC for the 38-mer assembly was 0.66±0.01. Cα coordinates of the model with optimal CCC (0.67) are provided as Dataset S2.

### Flexible fitting into cryo-EM maps

Flexible fitting was performed following the MDFF methodology [49] with NAMD 2.9. Symmetry restraints were employed for the prepore and pore conformation with the corresponding circular symmetries [50]. All MDFF simulations employed the CHARMM36 forcefield [51] *in vacuum* (long range interactions were cut off at 12 Å; dielectric constant of 80; 1 fs time-step; 298 K). Additional restraints were applied to preserve correct stereochemistry and prevent structural distortions [52] (secondary structure restraints force constant of 200 kCal mol^−1^ rad^−2^).

In each MDFF simulation minimization and equilibration steps were as follows: 10,000 steps of minimization with non-hydrogen atoms harmonically constrained, 100,000 steps of equilibration with protein main-chain constrained and 100,000 steps with C*α* atoms constrained. Three 5,000,000 steps MDFF runs were then performed with linearly increasing symmetry restraints to a final force constant of 5 kCal mol^−1^ Å^−2^ driving the system to the desired circular symmetry, and each with different grid force scaling parameter *ξ* = 0.2; 0.3; 0.5. Convergence was reached in all cases. Each of the three conformations obtained was subjected to 10,000 steps of minimization with *ξ* = 1.0. Therefore each starting conformation produced three final conformations.

### Pore model molecular dynamics simulation

A mixed square bilayer membrane patch was generated with DMPC and cholesterol (50/50 ratio, 1050 molecules each) with CHARMM-GUI 1.4 [53], [54] and the CHARMM36 forcefield [55] and equilibrated for 4 ns following the CHARMM-GUI provided settings in the presence of a TIP3P water layer of 15 Å thickness, 0.15 M sodium/chloride ions and planar constraints with the NAMD 2.9 software. The dimensions of the equilibrated bilayer system were 205 Å×205 Å×71 Å.

The tetramer pore model was energy-minimized for 2,500 steps free of cryo-EM restraints employing the Generalized Born/Solvent Accessible Surface Area implicit solvation [56]. Bilayer and solvent were then added and their height manually adjusted to match the position of the bilayer as judged from the cryo-EM density. Waters and lipids within 1.4 Å of the protein assembly were discarded. TIP3P waters were then added to a system of initial dimensions 205 Å×205 Å×175.5 Å. Ions were added to 0.15 M and system charge neutrality. The system was heated to 300 K and equilibrated in steps for 4 ns, first melting the lipid tails and cholesterol, then the headgroups and solvent and finally smoothly relaxing harmonic restraints on the tetramer. Care was taken to keep water molecules outside of the bilayer in the first steps of equilibration.

The system was then simulated for 15 ns in the NPAT ensemble free of constraints with PBC. Temperature was maintained at 300 K using Langevin dynamics with a damping constant of 1 ps^−1^. Pressure was maintained at 1 atm with a Nosé-Hoover-Langevin piston. An integration time step of 1 fs was used, short-range forces and long-range electrostatics were calculated every 1 and 2 fs respectively. Non-bonded interactions employed a 12 Å cut-off with a shorter Lennard-Jones switching function (11 to 12 Å) [55], long-range electrostatic forces were computed by the PME summation method (grid spacing smaller than 1 Å). The final dimensions of the system were 204 Å×204 Å×164 Å (709,660 atoms). Simulations were performed with the Multi-modal Australian ScienceS Imaging and Visualisation Environment (MASSIVE) [57].

## Supporting Information

Dataset S1 **PLY prepore model Cα coordinates.**
(PDB)

Dataset S2 **PLY pore model Cα coordinates.**
(PDB)

Figure S1 **Structural alignment illustrating the variability of CDC structures.** Conformers of PFO with a tight Domain2/TMH2 interface (see Table 2) are in blue, conformers with a weaker interface are in orange. ILY conformers are in green; ALO in red (both conformers are represented and have an overall rmsd <0.1 Å); SLO in yellow and SLY in teal.
(PDF)

Figure S2 **Domain 2 plasticity and Domain 4 flexibility in CDCs.** A. Left panel: distance of the Domain 2/TMH2 interface (see also Table 2). Center and right panels: values of twist at positions discussed in the text. The coloured vertical bars correspond to the range of twist values derived from the structural analysis. The pairs of residues considered are indicated at the top of each plot. The starting conformation for each MD simulation is indicated on the left of each panel. B. Each panel corresponds to an MD simulation whose starting conformation (cartoon representation, grey) is indicated at the left of the molecule. Cα positions taken from snapshots of the simulations are represented as dots after alignment on Domains 1–3 of each CDC molecule.
(PDF)

Figure S3 **Proposed conformational change involving residues of the β5 strand.** Coloring of C6 (pdb id: 3t5o) mimics CDC structurally equivalent positions.
(PDF)

Figure S4 **Monomer-Monomer β1–β4 Hydrogen bonds in the prepore model.** Mainchain atoms are represented in stick with one monomer in yellow and the adjacent monomer in blue. Dashed lines display the hydrogen bonds present in the model. The residues corresponding to the β5 strand of the yellow monomer are not displayed for clarity.
(PDF)

Figure S5 **Domain 4 residues exposure in the prepore conformation of PLY.** The position of residues is indicated by spheres at their Cα position. Asn402 (blue, Asn433 PFO numbering) was quenched by a collisional quencher in the prepore complex [9]. Lys395 (green, Lys426 PFO numbering) was not quenched. The membrane surface is defined by the cryo-EM map.
(PDF)

Figure S6 **Exposure and location of β-barrel forming residues.** A. Location of residues in the barrel overlaid with the cryo-EM density of the PLY pore. Residues in red have been determined to be located near the surface, in grey to be near the centre of the bilayer and in orange to be part of the hairpin turns [27]. B. Amphipathic pattern of membrane spanning amino-acids. Residues in grey have been determined to be exposed to the membrane bilayer, residues in blue have been determined to be exposed to the aqueous milieu [26], [27].
(PDF)

Figure S7 **Domain 4 residues exposure in the pore conformation.** Spheres at the position of their Cα indicate the position of residues. Only Domain 4 is shown. The residues shown are at position equivalent to PFO and in three categories: exposed (blue), interfacial (yellow) and buried (grey) as determined by Ramachandran et al. [29].
(PDF)

Figure S8 **Pore conformation in the context of representative CDC crystallographic structures and the prepore model.** The prepore model is in yellow, the pore conformation in pink. PFO I is in blue and ILY IA in green.
(PDF)

Figure S9 **Proximity of Domain 2 to adjacent subunits in the pore form.** The regions of potential interactions (red; Ala54-Asn56 and Thr384-Ser386, PFO numbering) are discussed in the text.
(PDF)

Figure S10 **Molecular dynamics simulation of the pore conformation.** A. Final snapshot of the simulation. The tetramer conformation is in cartoon presentation. Only the cholesterol oxygen (pink) and DMPC phosphate atoms (orange) are represented for clarity. The periodic box is depicted in grey. The regions indicated by arrows are discussed in the text. B. Tetramer rmsd plot versus simulation time.
(PDF)

Video S1 **Molecular dynamics simulation starting from PFO I (simulation 1).** The pair of relevant residues is represented as Cα spheres.
(MP4)

Video S2 **Molecular dynamics simulation starting from PFO I (simulation 2).** The pair of relevant residues is represented as Cα spheres.
(MP4)

Video S3 **Molecular dynamics simulation starting from PFO IIIA.** The pair of relevant residues is represented as Cα spheres.
(MP4)

Video S4 **Molecular dynamics simulation starting from PFO IIIB.** The pair of relevant residues is represented as Cα spheres.
(MP4)

Video S5 **Molecular dynamics simulation starting from PFO IIIC.** The pair of relevant residues is represented as Cα spheres.
(MP4)

Video S6 **Molecular dynamics simulation starting from ILY IA.** The pair of relevant residues is represented as Cα spheres.
(MP4)

Video S7 **Molecular dynamics simulation starting from ALO A.** The pair of relevant residues is represented as Cα spheres.
(MP4)
