# Supplementary figures and images for: A New Model for Pore Formation by Cholesterol-Dependent Cytolysins
Source: PLoS Comput Biol. 2014 Aug 21;10(8):e1003791. doi: 10.1371/journal.pcbi.1003791 (PMC4140638; doi:10.1371/journal.pcbi.1003791)

Representative  
structures

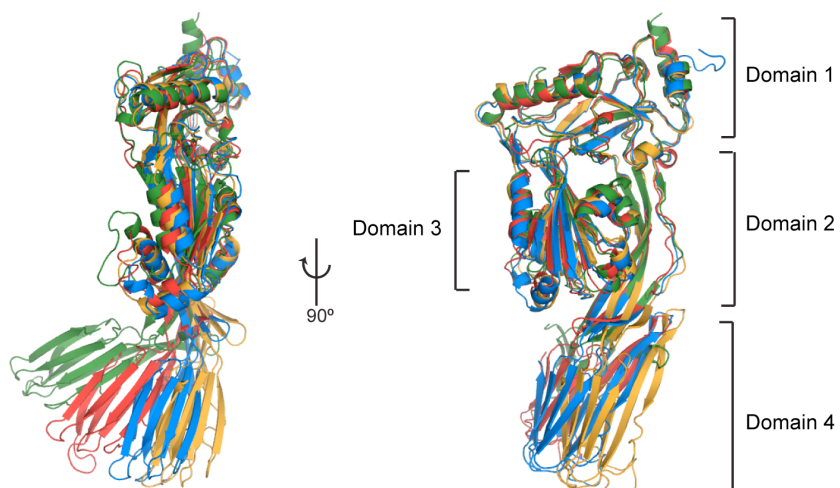

PFO

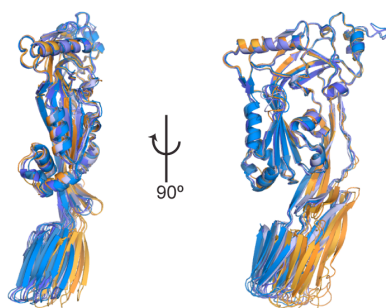

ILY

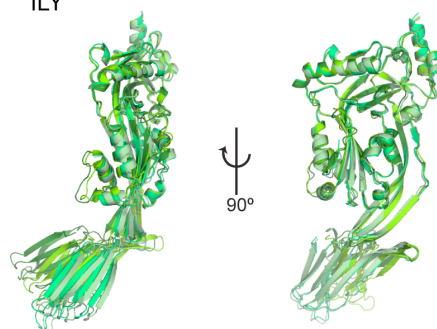

ALO

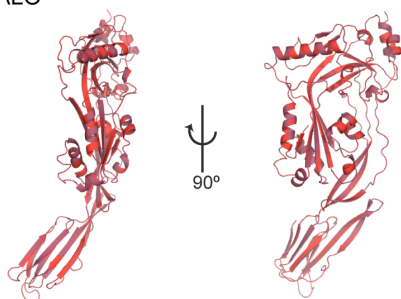

SLO & SLY

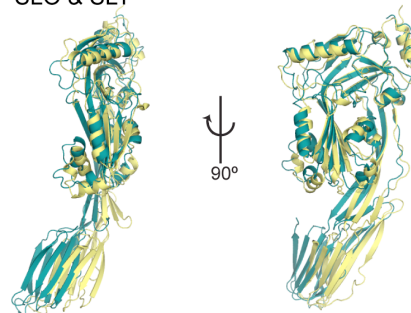

Supplement: Figure S1 — Structural alignment illustrating the variability of CDC structures. Conformers of PFO with a tight Domain2/TMH2 interface (see Table 2) are in blue, conformers with a weaker interface are in orange. ILY conformers are in green; ALO in red (both conformers are represented and have an overall rmsd <0.1 Å); SLO in yellow and SLY in teal. (PDF) [file pcbi.1003791.s003.pdf]

**A**

ILY IA

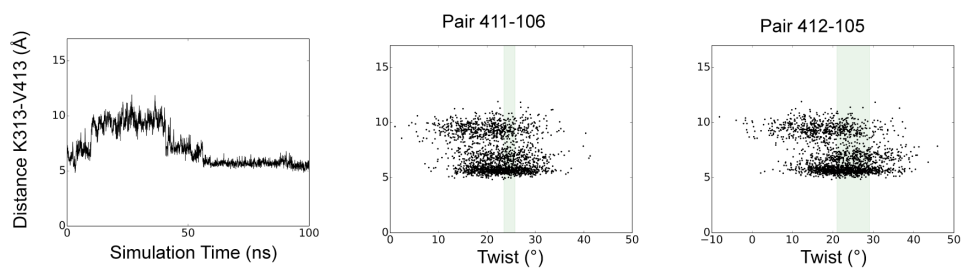

SLY

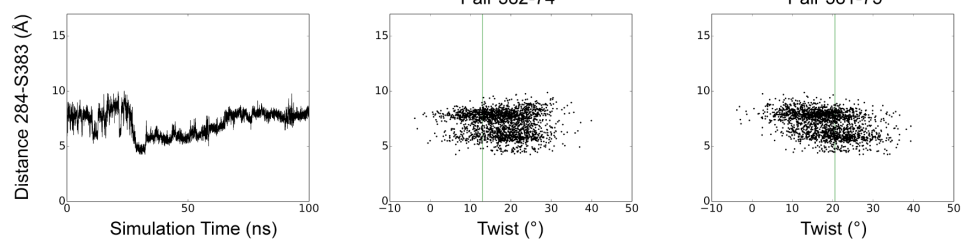

SLO

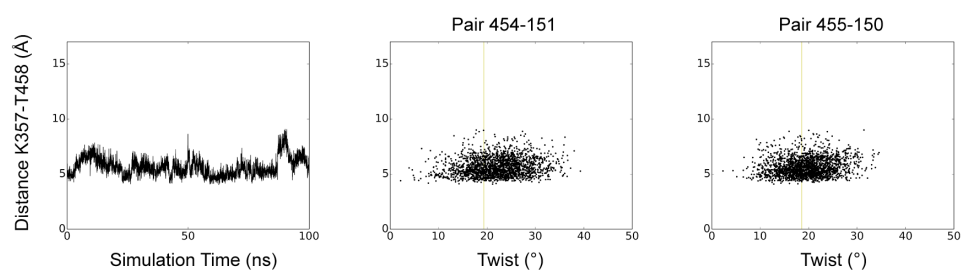

ALO A

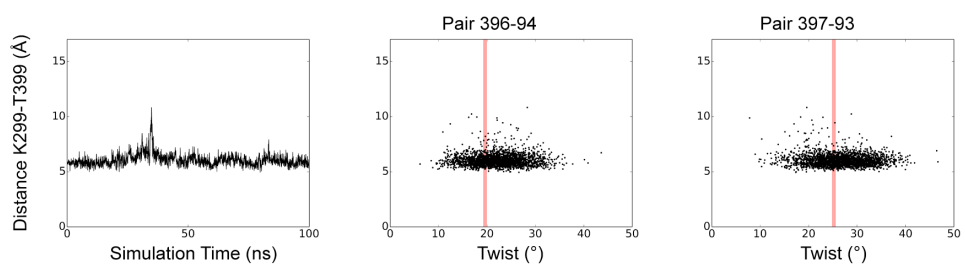

**B**

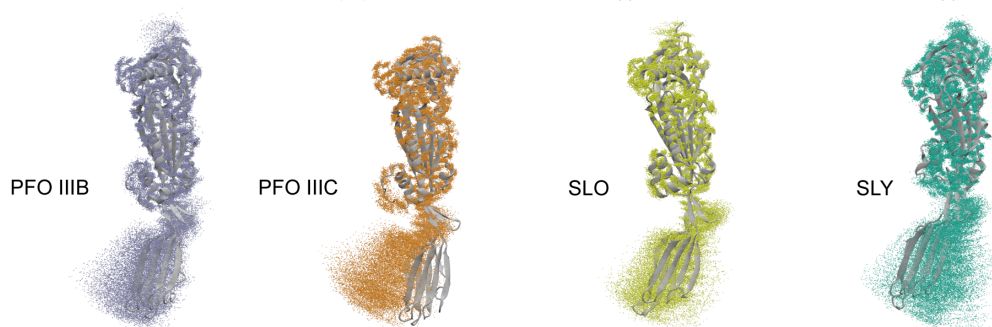

Supplement: Figure S2 — Domain 2 plasticity and Domain 4 flexibility in CDCs. A. Left panel: distance of the Domain 2/TMH2 interface (see also Table 2). Center and right panels: values of twist at positions discussed in the text. The coloured vertical bars correspond to the range of twist values derived from the structural analysis. The pairs of residues considered are indicated at the top of each plot. The starting conformation for each MD simulation is indicated on the left of each panel. B. Each panel corresponds to an MD simulation whose starting conformation (cartoon representation, grey) is indicated at the left of the molecule. Cα positions taken from snapshots of the simulations are represented as dots after alignment on Domains 1–3 of each CDC molecule. (PDF) [file pcbi.1003791.s004.pdf]

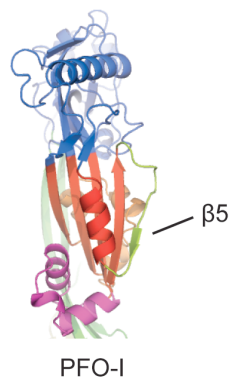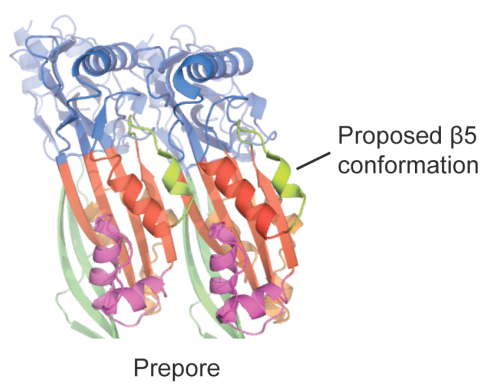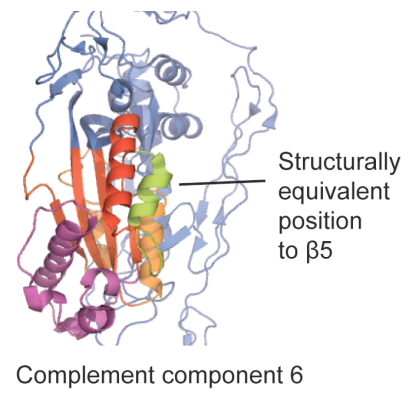

Supplement: Figure S3 — Proposed conformational change involving residues of the β5 strand. Coloring of C6 (pdb id: 3t5o) mimics CDC structurally equivalent positions. (PDF) [file pcbi.1003791.s005.pdf]

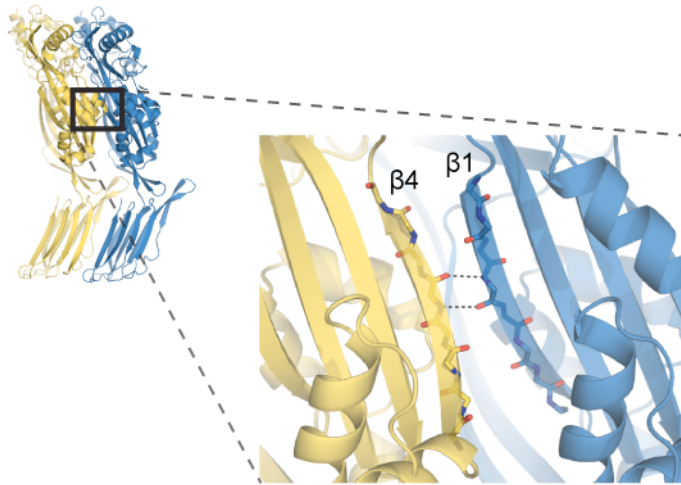

Supplement: Figure S4 — Monomer-Monomer β1–β4 Hydrogen bonds in the prepore model. Mainchain atoms are represented in stick with one monomer in yellow and the adjacent monomer in blue. Dashed lines display the hydrogen bonds present in the model. The residues corresponding to the β5 strand of the yellow monomer are not displayed for clarity. (PDF) [file pcbi.1003791.s006.pdf]

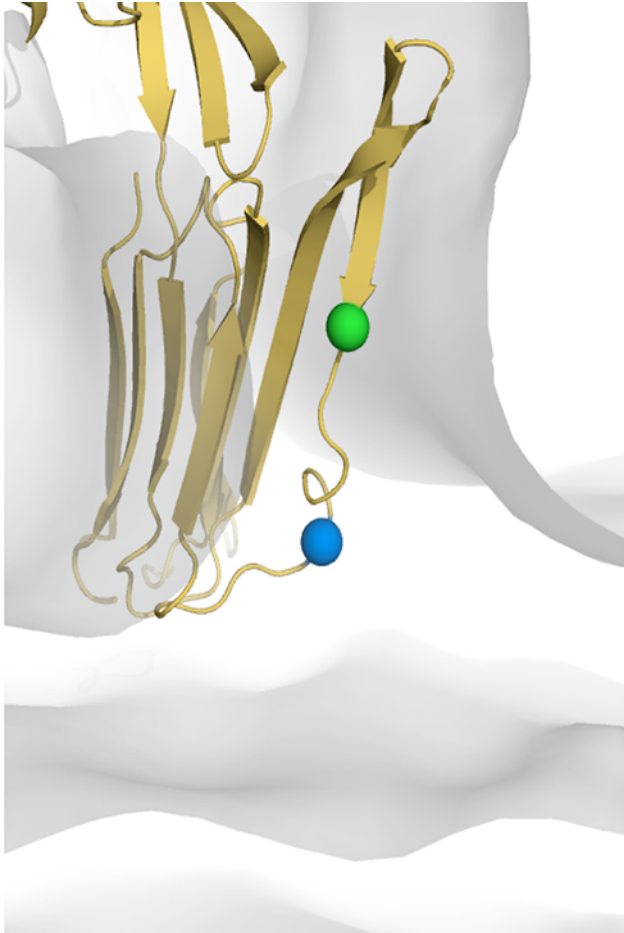

Supplement: Figure S5 — Domain 4 residues exposure in the prepore conformation of PLY. The position of residues is indicated by spheres at their Cα position. Asn402 (blue, Asn433 PFO numbering) was quenched by a collisional quencher in the prepore complex [9]. Lys395 (green, Lys426 PFO numbering) was not quenched. The membrane surface is defined by the cryo-EM map. (PDF) [file pcbi.1003791.s007.pdf]

**A**

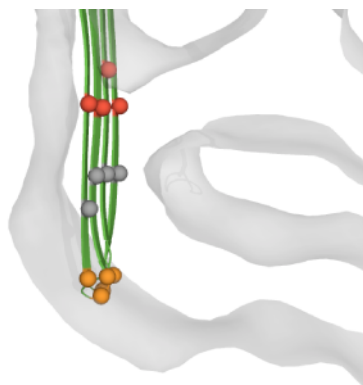

**B**

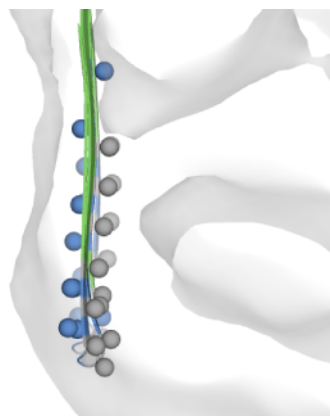

Supplement: Figure S6 — Exposure and location of β-barrel forming residues. A. Location of residues in the barrel overlaid with the cryo-EM density of the PLY pore. Residues in red have been determined to be located near the surface, in grey to be near the centre of the bilayer and in orange to be part of the hairpin turns [27]. B. Amphipathic pattern of membrane spanning amino-acids. Residues in grey have been determined to be exposed to the membrane bilayer, residues in blue have been determined to be exposed to the aqueous milieu [26], [27]. (PDF) [file pcbi.1003791.s008.pdf]

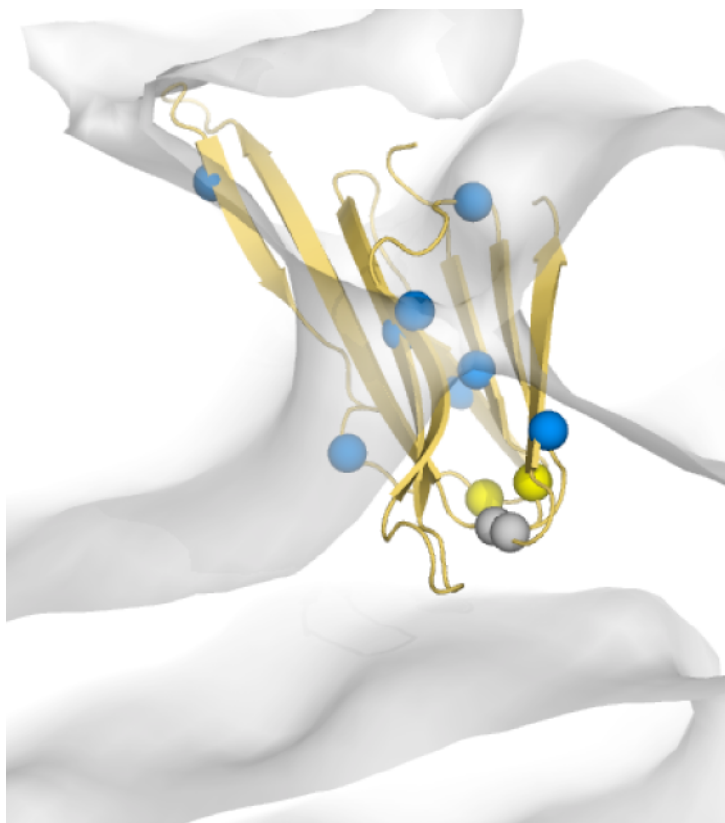

Supplement: Figure S7 — Domain 4 residues exposure in the pore conformation. Spheres at the position of their Cα indicate the position of residues. Only Domain 4 is shown. The residues shown are at position equivalent to PFO and in three categories: exposed (blue), interfacial (yellow) and buried (grey) as determined by Ramachandran et al. [29]. (PDF) [file pcbi.1003791.s009.pdf]

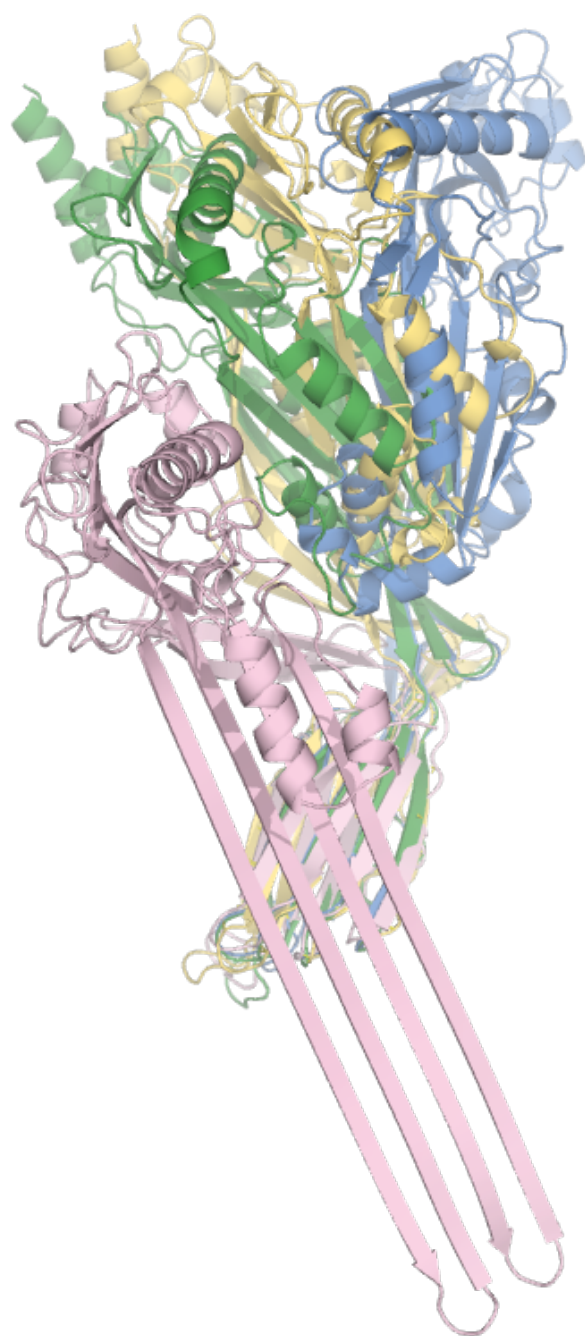

Supplement: Figure S8 — Pore conformation in the context of representative CDC crystallographic structures and the prepore model. The prepore model is in yellow, the pore conformation in pink. PFO I is in blue and ILY IA in green. (PDF) [file pcbi.1003791.s010.pdf]

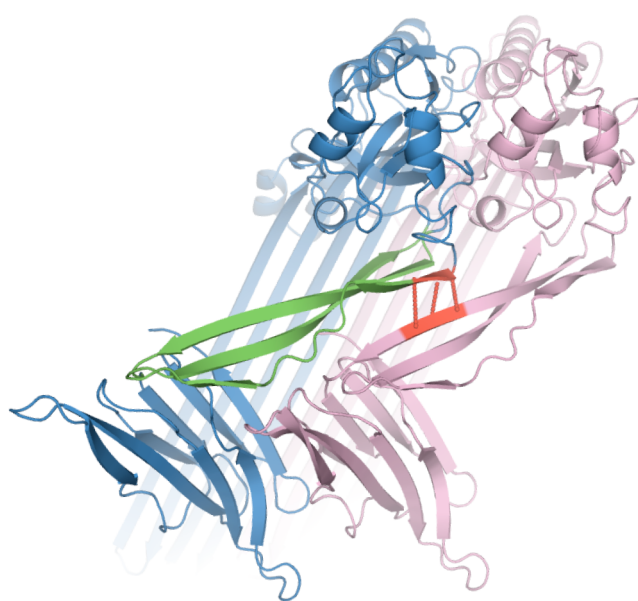

Supplement: Figure S9 — Proximity of Domain 2 to adjacent subunits in the pore form. The regions of potential interactions (red; Ala54-Asn56 and Thr384-Ser386, PFO numbering) are discussed in the text. (PDF) [file pcbi.1003791.s011.pdf]

**A**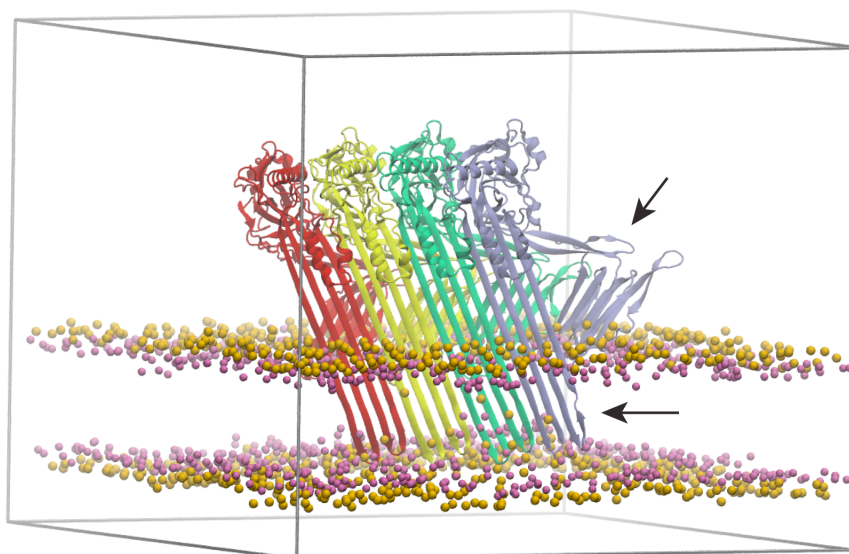**B**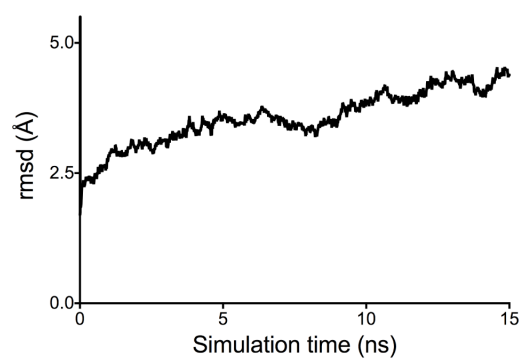

Supplement: Figure S10 — Molecular dynamics simulation of the pore conformation. A. Final snapshot of the simulation. The tetramer conformation is in cartoon presentation. Only the cholesterol oxygen (pink) and DMPC phosphate atoms (orange) are represented for clarity. The periodic box is depicted in grey. The regions indicated by arrows are discussed in the text. B. Tetramer rmsd plot versus simulation time. (PDF) [file pcbi.1003791.s012.pdf]
